# Caspase 3 as a Novel Marker to Distinguish Chromophobe Renal Cell Carcinoma from Oncocytoma

**DOI:** 10.1007/s12253-018-0548-8

**Published:** 2018-11-22

**Authors:** Adam Kowalewski, Łukasz Szylberg, Janusz Tyloch, Paulina Antosik, Izabela Neska-Długosz, Łukasz Frąckowski, Dominik Tyloch, Piotr Purpurowicz, Dariusz Grzanka

**Affiliations:** 1 Department of Clinical Pathomorphology, Collegium Medicum in Bydgoszcz, Nicolaus Copernicus University in Torun, ul. Sklodowskiej-Curie Str. 9, 85-094 Bydgoszcz, Poland; 2 Department of General and Oncologic Urology, Nicolaus Copernicus University, Bydgoszcz, Poland

**Keywords:** Chromophobe, ChRCC, Oncocytoma, Caspase 3, CASP3, Cyclin D1, p16, Survivin, CD138, Ki-67

## Abstract

Despite advances in our understanding of the biology of chromophobe renal cell carcinoma (ChRCC) and renal oncocytoma (RO), the differential diagnosis among these tumors remains one of the most problematic in renal pathology. Today, CK7 is the most recommended marker to distinguish these entities, however it appears insufficiently accurate by itself. This study aimed to find an easily accessible IHC stain that might out-compete CK7 in this field. Expressions of CK7, cyclin D1, p16, survivin, CD138, Ki-67 and caspase 3 (CASP3) were analyzed in a total of 27 cases (20 ROs and 7 ChRCCs). Immunoreactivity was assessed based on a combined score of the extent and intensity of staining. Compared to RO, a higher percentage of the total ChRCCs stained positive for CK7 (67% vs. 22%, respectively) and CASP3 (86% vs. 25%) (*P* < 0.005). The differences in staining with cyclin D1, p16, survivin, CD138 and Ki-67 turned out to be statistically insignificant in differentiating ChRCC from RO. CASP3 is a promising marker in distinguishing ChRCC from RO and may represent an alternative for CK7. Cyclin D1, p16, survivin, CD138 and Ki-67 cannot be used to distinguish these neoplasms.

## Introduction

Differential diagnosis among chromophobe renal cell carcinoma (ChRCC) and renal oncocytoma (RO) is one of the most problematic distinctions between renal neoplasms [1]. Despite the fact that numerous techniques for differentiating these two tumor histologies have been explored over the years, including histochemical stains, immunohistochemistry, chromosomal changes, molecular assays, and electron microscopy, we still encounter borderline cases [2]. Because oncocytoma is a benign disease, differentiating it from ChRCC is important in that it can often be treated conservatively [3]. ChRCC, on the other hand, is a serious condition and requires surgical removal [4, 5]. At present, histological features together with limited CK7 and Hale’s colloidal iron staining are most agreed upon for RO [2]. For tumors with mixed or inconclusive features, pathologists typically use the term “oncocytic renal neoplasm” with a comment that ChRCC cannot be completely excluded to be preferable to overdiagnosing a likely benign neoplasm as ChRCC [2].

To prevent such situations from happening and provide patients more accurate diagnosis, our team tried to find more suitable stain, which is also applicable in most pathology laboratories.

Heterogeneous nature of these two tumors constituted the starting point for the search. We presumed that ChRCC and RO might differ in proteins expressions proved to correlate with aggressiveness in other neoplasms.

We selected cyclin D1, p16, survivin, CD138, Ki-67 and caspase 3 (CASP3).

## Material and Methods

### Study Population

Pathologic records of 639 consecutive patients who underwent nephrectomy or nephron-sparing surgery for renal tumors between 2009 and 2017 were assessed in accordance with the Institutional Review Board guidelines. The study was performed in accordance with the Declaration of Helsinki.

We retrieved 24 cases of RO and 11 cases of ChRCC. All available hematoxylin and eosin (H&E) slides were reviewed and diagnosis was confirmed based on the presence of specific morphologic features. Vimentin and CD117 were used to exclude clear cell renal cell carcinoma and papillary renal cell carcinoma. Lesions that could not be diagnosed with the highest level of confidence were excluded. Ultimately, 20 cases of RO and 7 cases of ChRCC were enrolled in the study as the most representative sample. To minimize bias, clinicopathologic data such as sex, age, and tumor-node-metastasis stage are not mentioned.

### Immunohistochemistry

For immunohistochemistry, unstained 3 μm sections from FFPE tissue specimens were cut on the manual rotary microtome (AccuCut, Sakura, Torrance, USA). The immunohistochemical procedure was standardized using a series of positive and negative control reactions. Immunohistochemical stainings were performed according to well-known protocols [6, 7]. We investigated expressions of proteins: CK7, cyclin D1, p16, survivin, CD138, Ki-67 and cleaved CASP3.

For primary antibodies such as rabbit monoclonal cytokeratin 7 (SP52), rabbit monoclonal cyclin D1 (SP4-R), mouse monoclonal p16 (E6H4) and mouse monoclonal Ki-67 (30-9) (Ventana Medical Systems) immunohistochemical staining was performed on the Benchmark GX Platform (Ventana Medical Systems, Tuscon, AZ, USA). We used visualization system UltraView DAB IHC Detection Kit (Ventana Medical Systems, Tuscon, AZ, USA) as recommended by the producer. Using the EnVision system detection (Dako; Agilent Technologies, Inc., Santa Clara, CA, USA), immunohistochemical procedure was performed for monoclonal mouse Survivine (12C4), monoclonal mouse CD138 (MI15), (Dako; Agilent Technologies, Inc., Santa Clara, CA, USA) and CASP3 (Ab13847) (Abcam, Cambridge, UK).

### Scoring of Immunoreactivity

For each studied antibody, antigen expression evaluation was scored on a two-point scale: 0 - negative IHC reaction result, 1 – positive IHC reaction result. We consider such approach transparent and useful in routine diagnostics. Slides were examined in the light microscope ELIPSE E800 (Nikon Instruments Europe, Amsterdam, Netherlands) at 10x and 20x original objective magnification. Scoring was repetitively performed by three pathologists who were blinded to the clinical information.

### Statistical Analysis

All statistical analyses were performed using Statistica version 10 (StatSoft) and Microsoft Excel 2007. The comparative studies were analyzed statistically using the nonparametric chi-square test. The *P* value <0.05 was considered statistically significant.

## Results

**Fig. 1:**
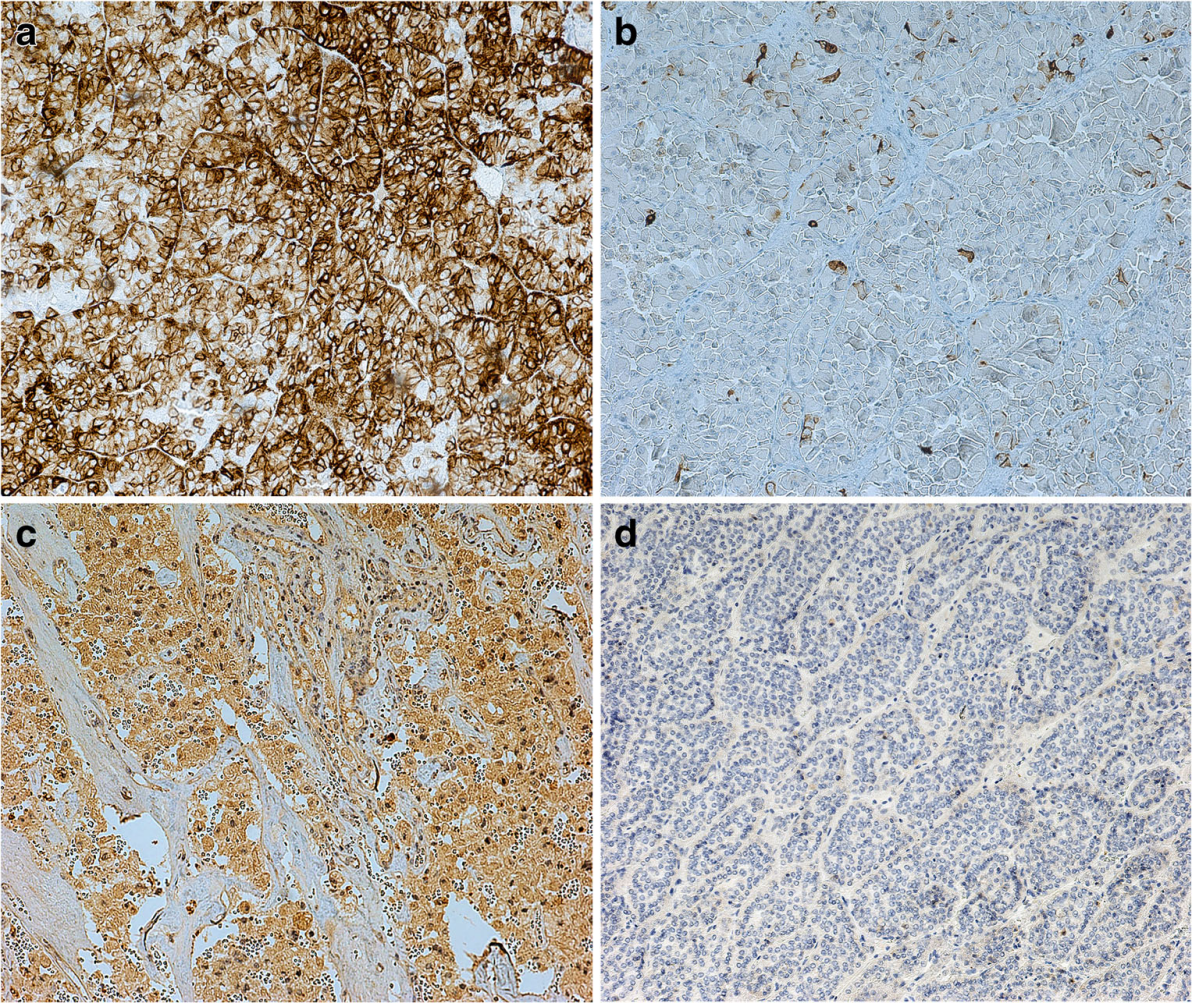
Immunohistochemical representative microphotographs show positive CK7 (**a**) and CASP3 (**c**) in ChRCC and negative CK7 (only scattered single positive cells) (**b**) and CASP3 (**d**) in RO. Primary objective magnification 10x. CASP3, caspase 3; ChRCC, chromophobe renal cell carcinoma; RO, renal oncocytoma

The immunohistochemical results of CK7, CASP3, cyclin D1, p16, survivin and CD138 in ChRCCs and ROs are reported in Table 1. 67% of ChRCCs revealed cytoplasmic positivity with membrane accentuation for CK7 whereas only 22% of ROs were positive for CK7 and revealed staining in only single cells (Fig. 1). The level of CK7 in ChRCCs was significantly higher than in RO group (*p* = 0.0047). CASP3 protein was strongly expressed in 75% of ChRCCs (Fig. 1) and the staining was cytoplasmic and selectively nuclear. Only 25% of RO were positive for CASP3. The level of CASP3 in ChRCCs was significantly higher than in RO group (*p* = 0.0001). Cyclin D1 expression was observed in 58% of ChRCCs and 38% of ROs. None of the ChRCCs were positive for survivin, whereas only 5% of ROs were positive. 17% of ChRCCs and 8% of ROs were positive for p16. CD138 positivity was found in 25% of ChRCCs and 24% of ROs. Cyclin D1, p16, survivin and CD138 did not reach statistical significance. Low number of ChRCC and RO cells were positive for Ki-67, 1% (±1.94) and *1%* (±1.37), respectively (Table 2)*.* CK7 and CASP3 help discriminate between ChRCC and RO. CASP3 showed 75% specificity, whereas the sensitivity was 86%. CK7 had high specificity (78%) and lower sensitivity (67%). Representative immunohistochemical staining is illustrated in Fig. 1.

**Table 1:** The immunohistochemical results of CK7, CASP3, cyclin D1, p16, survivin and CD138 in ChRCC and RO. CASP3, caspase 3; ChRCC, chromophobe renal cell carcinoma; RO, renal oncocytoma

Antigen	Kidney tumor	Immunopositive (%)	Immunonegative (%)	Statistical significance
CK7	RO	22	78	p = 0,0047
	ChRCC	67	33	
CASP3	RO	25	75	p = 0,0001
	ChRCC	86	14	
Cyclin D1	RO	38	62	p = 0,2125
	ChRCC	58	42	
p16	RO	8	92	p = 0,3947
	ChRCC	17	83	
Survivin	RO	5	95	p = 0,4043
	ChRCC	0	100	
CD138	RO	24	76	p = 0,9259
	ChRCC	25	75

**Table 2:** The immunohistochemical results of Ki-67 in ChRCC and RO. ChRCC, chromophobe renal cell carcinoma; RO, renal oncocytoma

Antigen	Kidney tumor	Median	Mean Avarage	Std. Deviation	Statistical significance
Ki-67	RO	1	1,34	1,37	p = 0,555
	ChRCC	1	1,83	1,94	

## Discussion

Overlapping morphologic characteristics of RO and ChRCC pose a diagnostic challenge. Hence, a range of methods emerged as an adjunct to conventional H&E staining.

Electron microscopy was performed in the past. Now it is found time-consuming and too expensive. Genetic testing, although not available in most facilities [8, 9], may be used to confirm a diagnosis of numerous neoplasms, including RO and ChRCC. Using next-generation sequencing (NGS), Durinck et al. analyzed exome, transcriptome, and copy number alteration data from primary renal tumors. Analysis of differential expression in ChRCC versus RO identified a minimal set of 5 genes - ASB1, GLYAT, PDZK1IP1, PLCG2 and SDCBP2 - that were sufficient to separate ChRCC from RO [10]. In addition to widespread differences in gene expression between ChRCC and RO, unsupervised clustering of mRNA profiles indicated further molecular heterogeneity within ChRCC, with at least two subsets identified [11]. Gerlinger et al. detected expression signatures of good or poor prognosis in different regions of the same renal tumor [12]. It may account for the benefits associated with cytoreductive nephrectomy [13, 14] that would eliminate an evolutionary reservoir of phenotypic tumor-cell diversity. This is why particularly renal intratumor heterogeneity leads to underestimation of the tumor genomics landscape portrayed from single tumor-biopsy samples and presents major challenges to personalized-medicine and IHC markers development [12]. On the other hand, identification of the most accurate stain for separation ChRCC from RO may help reconstruct the trunk of the phylogenetic tree and also contribute to novel therapeutic approaches.

Hale colloidal iron stain, despite its specificity, is technically demanding and frequently difficult to assess. Multitude of markers including CK7, kidney-specific cadherin, CD10, EMA, RCC, MOC31, S100A1, parvalbumin and RON proto-oncogene have been used to distinguish ChRCC from RO, however no single marker appears sufficiently accurate by itself [8, 15]. For this reason panels of markers were suggested [9, 16]. Although the panels achieved higher sensitivity and specificity than any marker alone, they did not become as popular as CK7 in this field [2].

In ChRCC there is a strong and diffuse staining for CK7, comparing to scattered single cells in RO [1]. Five studies are compatible with this statement [9, 17–20]. Remaining authors reported lower sensitivity and specificity of this marker [21–23]. They indicated that positive CK7 immunostaining starts from 63% of ChRCC patients [24], while scattered single positive cells might be observed in only 4% of RO cases [25]. The conflicting results could be caused by the variance in pathologic diagnosis, tumor heterogeneity, use of different antibodies and reagents for the studies, and different laboratory staining procedures. Some of CK7 negative cases may be better classified as oncocytic renal neoplasm unless EM proves that they are ChRCC. Our results support the utility of CK7, however its low sensitivity (67%) discourages from using it as the only stain. Nevertheless, CK7 staining remains the most recommended in differential diagnosis between ChRCC and RO [1]. That indicates the growing need of novel, reliable and easily accessible marker that individually or together with histologic findings solves the dilemma.

Cyclin D1 was considered promising due to its role in progression through the G1 phase of the cell cycle. Deregulation of cyclin D1 not only promotes mitogen-independent proliferation, but also affects other cellular processes, both directly and indirectly, in ways that have potentially oncogenic consequences [26]. Cyclin D1 was highly expressed in close to half of ROs (38%) and ChRCCs (58%) suggesting its complex role in biology of both of these tumors. Nevertheless, its role in distinguishing both tumors is unsatisfactory.

p16 contributes to the regulation of cell cycle progression by inhibiting the S phase, and is one of the main factors to avert tumor formation. Close to 50% of all cancers show p16 inactivation. Intriguingly, overexpression of p16 has also been described in several tumors [27]. Most of ROs and ChRCCs were p16 negative indicating that their cells similarly evade the senescence.

Survivin is involved in inhibition of apoptosis and regulation of cell cycle. Expression pattern of survivin is distinctive; it is prominently expressed during embryonal development, absent in most normal, terminally differentiated tissues but upregulated in a variety of cancers. Expression of survivin correlates with aggressiveness of tumors [28]. According to our results, survivin plays no significant role in biology of RO, nor ChRCC.

CD138 (syndecan-1) is well known to be associated with cell proliferation, adhesion, and migration in various malignancies. CD138 promotes angiogenesis in ductal breast carcinoma [29] and correlates with urothelial cancer recurrence [30]. CD138 appeared highly expressed in one fourth of both ROs and ChRCCs and cannot be used to distinguish these neoplasms.

ROs and ChRCCs differ in growth kinetics. Richard et al. reported that annual growth rate was 0.14 cm for RO and 0.38 cm for ChRCC [31]. Expression of cell proliferation marker such as Ki-67 might reflect these differences. The fraction of Ki-67-positive tumor cells is often correlated with the clinical course of cancer [32]. The differences in staining with Ki-67 turned out to be statistically insignificant in distinguishing ChRCC from RO.

CASP3 is a cysteine-aspartic acid protease that plays a central role in the execution-phase of cell apoptosis. Although CASP3 activation causes cell death in the host cell, it has been found to stimulate cell proliferation in neighbouring, non-apoptotic cells [33]. Fang et al. named this mechanism the “Phoenix Rising” pathway, as it is a manifestation of the inextricable link between the Yin and Yang of cellular life and death in metazoan organisms. This phenomenon is important in wound healing and tissue regeneration [34], however it may potentially hinder success in cancer therapy. Emerging evidence has indicated that apoptotic tumor cells stimulate the repopulation of tumors from a small number of surviving cells by CASP3 regulation. High levels of activated CASP3 significantly correlate with a poor prognosis in a number of cancers [33, 35–37]. Distinct outcomes in patients with RO and ChRCC might be a result of different CASP3 expression accordingly.

Both RO and ChRCC share a common origin from intercalating cells of the distal renal tubules [38, 39]. Their common coexistence causes difficulty to render a diagnosis basing on limited tissue biopsy samples. As a general rule, if the lesion resembles ChRCC on needle biopsy, it can be confidently reported as such. By comparison, a lesion resembling RO may be incompletely sampled, with other areas merging into the ChRCC. In fact, it may be a hybrid oncocytic/chromophobe tumor or oncocytoma-like areas in ChRCC [8, 40]. The existence of a hybrid together with similarity of both neoplasms speak for the concept that these entities might belong to the same morphologic spectrum [41]. Perhaps under certain circumstances ChRCC may actually arise from RO and alteration in CASP3 expression plays a pivotal role in the malignant transformation. That would explain high sensitivity and specificity of this marker.

## Conclusions

CK7 does not show the satisfactory immunoreactivity in ChRCCs and ROs. Meanwhile, a very high level of diagnostic certainty is required if surgical intervention is to be avoided. We propose CASP3 as a reliable and also easily accessible marker. Our results show that CASP3 fits the optimal diagnostic strategy of separation ChRCC from RO (Table 3). Further studies are needed to investigate this approach.

**Table 3:** Differentiating features of ChRCC and RO. ChRCC, chromophobe renal cell carcinoma; RO, renal oncocytoma; −, negative; +, positive

Features	RO	ChRCC
Micro description	Nests floating in hypocellular stroma	Diffuse, sheet like pattern
	Clear cells only focally in hyalinized scars	Clear cells usually prominent, frequently peripheral in nests
	–	Perinuclear halos
Histochemistry	Hale colloidal iron (−)	Hale colloidal iron (+)
Immunohistochemistry	CASP3 (−); CK7 (−)	CASP3 (+); CK7 (+)
